# Health Risk Assessment of PAHs from Estuarine Sediments in the South of Italy

**DOI:** 10.3390/toxics11020172

**Published:** 2023-02-13

**Authors:** Fabiana Di Duca, Paolo Montuori, Ugo Trama, Armando Masucci, Gennaro Maria Borrelli, Maria Triassi

**Affiliations:** 1 Department of Public Health, University “Federico II”, Via Sergio Pansini n° 5, 80131 Naples, Italy; 2 General Directorate of Health, Campania Region, Centro Direzionale Is. C3, 80143 Naples, Italy

**Keywords:** polycyclic aromatic hydrocarbons (PAHs), river sediment, occurrence, incremental lifetime cancer risk, carcinogenic risk

## Abstract

Increased concerns about the toxicities of Polycyclic Aromatic Hydrocarbons (PAHs), ubiquitous and persistent compounds, as well as the associated ecotoxicology issue in estuarine sediments, have drawn attention worldwide in the last few years. The levels of PAHs in the Sele, Sarno, and Volturno Rivers sediments were evaluated. Moreover, the cancerogenic risk resulting from dermal and ingestion exposure to PAHs was estimated using the incremental lifetime cancer risk (ILCR) assessment and the toxic equivalent concentration (TEQ_BaP_). For Sele River, the results showed that the total PAH concentration ranged from 632.42 to 844.93 ng g^−1^ dw, with an average value of 738.68 ng g^−1^ dw. ∑PAHs were in the range of 5.2–678.6 ng g^−1^ dw and 434.8–872.1 ng g^−1^ dw for the Sarno and Volturno River sediments, respectively. The cancerogenic risk from the accidental ingestion of PAHs in estuarine sediments was low at all sampling sites. However, based on the ILCR_dermal_ values obtained, the risk of cancer associated with exposure by dermal contact with the PAHs present in the sediments was moderate, with a mean ILCR_dermal_ value of 2.77 × 10^−6^. This study revealed the pollution levels of PAHs across the South of Italy and provided a scientific basis for PAH pollution control and environmental protection.

## 1. Introduction

Estuaries are the main deposits for the disposal of industrial and domestic effluents, sewage sludge, and dredged material with a significant load of contaminants, including PAHs, from pipeline discharges, vehicular emissions, atmospheric deposition, surface runoff, as well as oil spills in aquatic environments [1]. Due to their low water solubility and high lipophilicity, PAHs tend to accumulate in the sediments of aquatic systems for long periods due to their high degradation resistance and high organic carbon content [2]. In soil and sediment compartments, PAHs can undergo biodegradation processes by microorganisms. However, due to their stable physic–chemical characteristics, hydrophobicity, and a strong tendency to absorb into the soil matrix, their biodegradation rate is low. Consequently, PAHs do not degrade easily, and they can accumulate in the solid phase of the terrestrial and aquatic environment, where they persist for a long time [3,4]. Thus, sediments constitute a natural reserve of PAHs in the aquatic system [5,6]. Moreover, they can be released into the surrounding environment by means of resuspension phenomena, thus giving rise to “secondary pollution”. Consequently, since high concentrations of PAHs in sediments may reveal a potential pollution risk for the environment and human health, it is essential to monitor these compounds in sediment to protect and preserve the aquatic environment and human health [7].

PAHs, as persistent organic pollutants (POPs), are widely present in the ecosystem [8,9,10]. Their spread to the environment has raised many concerns for human health, as some of them have been identified as carcinogens, mutagens, and teratogens. Indeed, PAHs released into the environment may enter the food chain, and exposure to them may result in a risk of cancer or other adverse effects on human health [11,12,13]. The International Agency for Research on Cancer (IARC) has classified PAHs according to their carcinogenicity as carcinogenic (Group 1), probable carcinogenic (Group 2A), possible carcinogenic (Group 2B), and non-carcinogenic (Group 3) [14]. In general, high molecular weight PAHs (4–6 rings) are more toxic than low molecular weight PAHs (2–3 rings) [15]. The greater toxicity of the former is due to the greater number of aromatic rings from which dihydrodioloepoxides are formed [16]. In particular, the fat solubility of PAHs makes them dangerous because they can cross cell membranes, penetrate, and deposit in tissues. In tissues, PAHs can be oxidized to epoxide (where an oxygen atom replaces one of the double bonds C=C) by a monooxygenase associated with cytochrome P 450 present in the endoplasmic reticulum of cells. The epoxide thus formed can attack macromolecules such as DNA, hence the mutagenic and carcinogenic action of the PAHs, or be transformed into diol by enzymatic systems such as epoxide hydrolase (EH). This reaction of detoxification allows the formed diol, with two hydrophilic alcohol groups, to be more soluble than the starting compound and then to be expelled from the body more easily [17]. In fact, Lee et al. reported that cytochrome P450 enzymes can metabolize BaP and activate it into a carcinogenic reactive intermediate or metabolite. Consequently, these substances can bind to DNA, resulting in DNA adducts that interfere with DNA replication, causing cytotoxicity, teratogenicity, genotoxicity, immunotoxicity, mutagenesis, and carcinogenesis [18].

Mainly, Benzo[a]pyrene (BaP) has been classified as genotoxic using in vitro tests and in vivo studies. In laboratory animals, oral administration of BaP induced tumors of the stomach and mammary gland and skin cancer [19,20]. According to the WHO, BaP is the compound with the greatest negative consequences for human health and has been included in Group 1, which includes all those substances for which there is sufficient evidence of carcinogenicity in humans [20]. BaP has been classified as genotoxic [10], and it has been involved in tumor development in all test animal species tested, regardless of the route of exposure (oral, cutaneous, subcutaneous, inhalator, intratracheal, intrabronchial, intraperitoneal, or intravenous) [21]. Furthermore, dibenz[a,h]anthracene (DahA) has been classified as a probable carcinogen and/or mutagen for humans, and it is included in Group 2A unlike Benzo[a]anthracene (BaA), Benzo[b]fluoranthrene (BbF), Benzo[k]fluoranthrene (BkF), Chrysene (Chr), and Indeno [123-cd]pyrene (IcdP), which have been included in Group 2B as possible carcinogens in humans [22].

The main routes of exposure to PAHs in the general population are inhalation from breathing ambient and indoor air or smoking cigarettes, ingesting food containing PAHs, and breathing smoke from open fireplaces [10,23,24]. According to Adeniji et al., exposure via inhalation, ingestion, or skin contact may lead to human health problems resulting from short- and long-term effects, including some serious respiratory and cardiovascular diseases [13].

The acute effects of PAHs on human health mainly depend on the duration of exposure, PAH concentration during exposure, and the toxicity of the compounds to which one is exposed, as well as the route of exposure. Short-term exposure to PAHs has been reported to cause impaired lung function such as asthma and thrombotic effects in people with coronary heart disease [24]. However, there is currently no full understanding of the effects of short-term exposure to PAHs, but it is well-known that occupational exposure to high levels of PAH-containing mixtures causes symptoms such as eye irritation, nausea, and vomiting [25]. Moreover, PAHs mixtures are also known to cause skin irritation and inflammation, as Anthracene (Ant), Benzo[a]Pyrene (BaP), and Naphthalene (NaP) are skin irritants [26]. In addition, PAHs interfere with hormonal systems and, as a result, can have harmful effects on reproduction and immune function [27]. The adverse effects of exposure to PAHs have been extensively investigated, but the information currently available on human exposure to individual PAHs is scattered and incomplete, except for some accidental contact with NaP and BaP [28,29,30]. Srogi et al. stated that prolonged dermal contact with NaP may cause redness and inflammation of the skin [31]. In addition, Diggs et al. reported that long-term exposure to low levels of Pyr and BaP has been identified as the cause of cancer in laboratory animals [32]. Animal studies have also shown adverse effects on reproduction and development due to exposure to PAHs, whereas these effects were not commonly detected in humans [33,34]. Moreover, Anyahara stated that exposure to PAHs can induce cataracts and cause kidney and liver damage and jaundice [35].

Estuaries are important aquatic systems largely affected by PAH pollution. In fact, due to their chemical properties, PAHs can persist in water where they are readily adsorbed onto particulate matter, settling in river sediments and soils. Thus, river sediments behave as the primary sink and reservoir for PAHs in the aquatic environment [36,37]. Consequently, sediments are important indicators since they can reflect the pollution status of the environment [38].

To date, no previous studies have evaluated the carcinogenic risk to human health associated with dermal and accidental ingestion exposure to PAHs from surface sediments in the South of Italy. Therefore, the main aim of this study was to assess the risk to human health from exposure to PAHs present in the sediments of surface waters in a large coastal area of the Campania Region, in the South of Italy. Specifically, the purpose of this study was to evaluate the distribution patterns of PAHs and to assess the carcinogenic risk to human health from dermal and ingestion exposure to these contaminants from the estuarine sediments of the Sarno, Volturno, and Sele Rivers, which are the main surface water streams of the Campania Region, in the South of Italy.

## 2. Materials and Methods

### 2.1. Study Area

**Figure 1:**
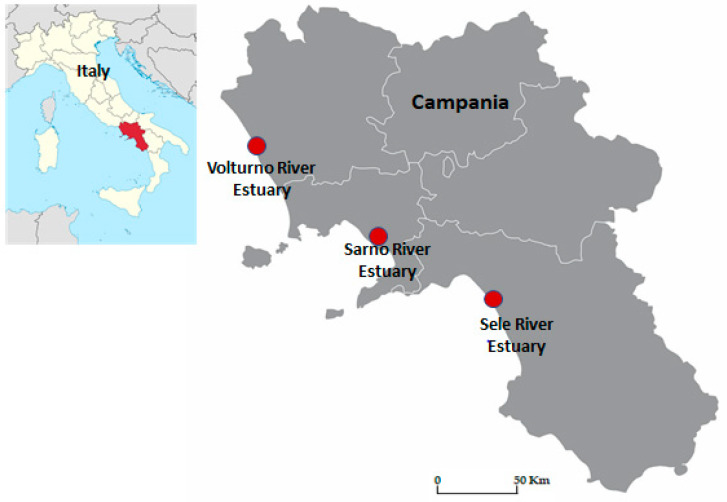
Study area.

This assessment of the human health risk from exposure to PAHs was carried out in a study area of approximately 3100 km^2^ and included the three largest plains in the Campania Region, in the South of Italy. Particularly, the research area was close to the estuaries of the Sele, Volturno, and Sarno Rivers, which traverse the same-named plains. Figure 1 shows the three plains of interest and the respective estuaries of the rivers that cross them.

### 2.2. Sampling

**Figure 2:**
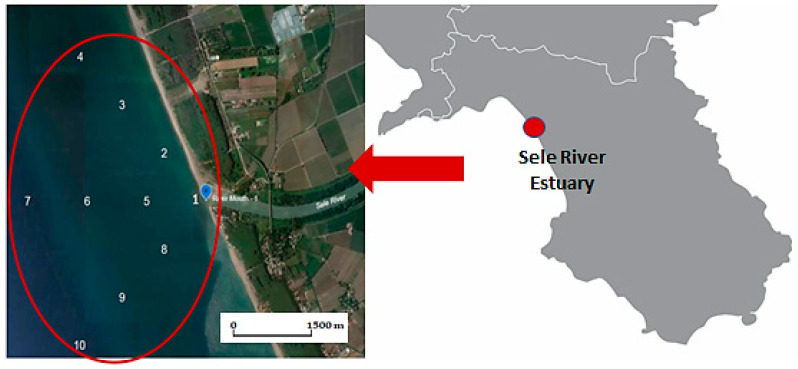
Hydrographic network and sampling sites near the Sele River.

A sampling campaign was carried out during the spring season (April 2021) at 10 sampling sites near the Sele River Estuary. In detail, sediment samples were taken from the mouth of the river (Site 1) at different distances, 500 m, 1000 m, and 1500 m from the mouth, and directions, to the north, west, and south of the estuary (Figure 2). During sampling, a global positioning system (GPS) was used to locate all sampling sites. Information on the identification number (ID), characteristics, and coordinates of each sampling location are shown in Table 1. The samples were collected at a depth of 0 to 5 cm using a scraping sampler (Van Veen Grab) and placed in aluminum containers. Then, they were transferred under refrigeration to the laboratory and stored at −20 °C until analysis.

**Figure 3:**
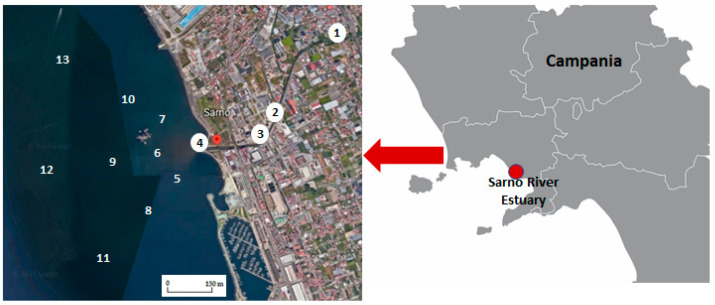
Hydrographic network and sampling sites near the Sarno River.

The PAHs levels in sediment samples from the Sarno and Volturno Rivers were evaluated previously [39,40]. Briefly, for the Sarno River, a sampling campaign was carried out during the spring of 2008 at the source of the river (site 1), just before and after the junction with Alveo Comune, at the river mouth (site 4), and in 9 sites located at different distances from the estuary (Figure 3). More detailed information about the sediment sampling in the Sarno River is given in Table 2.

**Figure 4:**
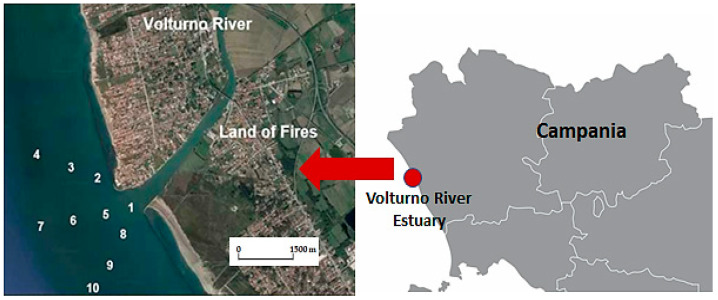
Hydrographic network and sampling sites near the Volturno River.

For the Volturno River, the sampling campaign was carried out in April 2018 near the mouth of the Volturno River and in 9 sites located at different distances from it (Figure 4). The specific details are provided in Table 3.

### 2.3. Extraction Procedure and Clean-Up

The analyses were performed as described previously [41]. Briefly, for PAH extraction, the sediment samples were air-dried, crushed, sieved in 250 µm particles, and then divided into portions of 5 g. The PAH concentrations were indicated as dry weight (ng/g dw) [42,43]. The PAH extraction was performed with a Soxhlet extractor using methylene chloride as solvent. Subsequently, the extracts, first purified using a column composed of sodium sulfate/silica gel and then eluted with 70 mL of a hexane:methylene chloride (7:3, *v*/*v*) solution, were evaporated to dryness and reduced to a final volume (500 μL) with the aid of a weak current of nitrogen. Finally, the extracts were analyzed using gas chromatography coupled with mass spectrometry (GC-MS). A TOC analyzer was used to evaluate the total organic carbon (TOC) content in the sediment samples (TOC-VCPH, Shimadzu Corp., Kyoto, Japan).

### 2.4. Instrumental Analysis, Quality Assurance, and Quality Control

A TRACE^TM^1310 gas chromatograph coupled to an ISQ^TM^7000 single quadrupole mass spectrometer (GC-MS, Thermo Scientific, Waltham, MA, USA) was used, equipped with a capillary column TG-5MS (length 30 mm, inner diameter 0.25 mm, film thickness 0.25 μm) and helium as a gas carrier (constant flow of 1 mL/min), operating in the electronic ionization mode (EI) set to 70 eV. The injector operated at 280 °C, and the temperature of the detector was set to 300 °C. The acquisition was performed with the Selected Ion Monitoring (SIM) mode using two characteristic fragments for each selected analyte. A splitless injection mode was adopted with an injection volume of 1 μL. The quantification of PAHs was carried out using response factors related to the respective internal standards based on a six-point calibration curve for individual PAHs (Dr. Ehrenstorfer GmbH, Augsburg, Germany) (R^2^ > 0.97). Chrysene-d_12_ was used as an internal standard for sample quantification. Before the analysis, all the glassware to be used was thoroughly washed with methanol, acetone, and dichloromethane and placed in the oven at 200 °C to minimize possible sources of contamination. The column temperature was set with different gradients: from 60 °C to 200 °C with an increase of 25 °C/min (kept for 2 min), to 270 °C increasing at 10 °C min^−1^ (kept for 6 min), and to 310 °C with a rise of 25 °C min^−1^ (kept for 10 min). The single ion monitoring mode (SIM) was used for the acquisition using characteristic ions for each target analyte. The 16 priority IPA, according to the WHO and USEPA, were evaluated (Table 4) [20,44].

Six-point calibration curves (5–10–50–250–500–1000 ng/L), procedural blanks, and sample triplicates were carried out for every set of samples. The PAH concentrations were calculated as dry weight (ng/g dw). The limits of detection (LOD) and quantification (LOQ) were evaluated as three and ten times the noise in blank samples, respectively. They were in the range of 1.5–1.9 ng g^−1^ and 5.1–6.3 ng g^−1^, respectively. In the procedural blanks, analyzed as the samples, the PAHs showed a concentration below the LOD. Moreover, for individual PAHs, the recovery test values ranged from 80% to 97%, meeting the quality control criteria (70–130%). For the effective and reproducible detection and quantification of low concentrations of PAHs in sediments, the linear range, precision, limits of detection, and limits of quantification were performed. The precision of the method was determined using repeatability tests and was expressed as standard deviation (SD) (Tables S1–S3). The average of the results was used to estimate the precision of the method.

### 2.5. Human Health Risk Assessment

The human health risk assessment is useful in determining whether exposure to a chemical in a specific dose may cause an increase in the frequency of adverse effects on human health [39,42,45,46,47]. For the population, PAH exposure represents a health and hygiene risk, which is assessed as a carcinogenic risk. Therefore, the USEPA defined carcinogenic risk as the probability that an individual may develop cancer over a lifetime from exposure to a specific substance classified as mutagenic or carcinogenic. Thus, this risk assessment consists of two phases, which are the estimation of the probability of an event occurring and the study of the likely magnitude of its adverse effect over a specific time frame [48].

The incremental lifetime cancer risk (ILCR) due to exposure by direct ingestion and skin contact to PAHs present in the sediment was evaluated [49]. First, the doses of contaminants taken up by human receptors through the two different exposure pathways considered were calculated according to Equations (1) and (2) [50]:

(1) $${\mathrm{Dose}}_{\mathrm{ing}}=\frac{{\;C}_{s\;}\times {\;\mathrm{IR}}_{s}\times {\mathrm{RAF}}_{\mathrm{oral}}\times D_{\mathrm{hours}}\times D_{\mathrm{days}}\times D_{\mathrm{weeks}}\times {\mathrm{ED}}_{\mathrm{years}}}{\mathrm{BW}\times \mathrm{LE}}\;$$

(2) $${\mathrm{Dose}}_{\mathrm{derm}}=\frac{C_{s}\times {\mathrm{SA}}_{h}\times {\mathrm{SL}}_{h}\times {\mathrm{RAF}}_{\mathrm{derm}}\times \mathrm{EF}\;\times D_{\mathrm{days}}\times D_{\mathrm{weeks}}\times {\mathrm{ED}}_{\mathrm{years}}}{\mathrm{BW}\times \mathrm{LE}}$$

where:

Dose_ingestion_ (mg/kg-day) indicates the dose from accidental sediment ingestion; Dose_dermal_ (mg/kg-day) is the dose from skin contact with sediment; C_s_ (mg/kg) represents the concentration of the contaminant in the sediment; *IR_s_* (kg/day) is the rate of accidental sediment ingestion; RAF_oral_ indicates the relative absorption factor for the gastrointestinal tract; RAF_derm_ (dimensionless) expresses the relative absorption factor for the skin. Moreover, the dose was evaluated based on the hours per day with exposure: 0–16/16 h for accidental ingestion of sediment (D_hours_); days in a week with exposure [(0–7)/7 days] (D_days_); weeks in a year with exposure [(0–52)/52 weeks] (D_weeks_); total years with exposure (ED_years_); surface of hands (assuming only hands are exposed) (SA_h_ (cm^2^); SL_h_ (kg/cm^2^-event) = Sediment load rate on exposed skin; EF (event/day) = Number of skin exposures per day; BW (kg) = receptor body weight; LE = life expectancy/average life expectancy expressed in years; CF (conversion coefficient) = 1 × 10^−6^ kg/mg.

Additionally, since Benzo(a)pyrene (BaP) is the most carcinogenic compound among the PAHs considered [10], all individual analyte concentrations were converted to the corresponding toxic equivalent concentrations of BaP. These concentrations are referred to as TEQ_BaP_ or BaP_eq_ and were obtained using the concentration product for toxic equivalence factor (TEF). The TEF factors for the 16 US EPA priority PAHs are shown in Table 5 [51].

The total concentrations of PAHs were obtained using the sum of the calculated toxic equivalents for each compound in relation to BaP. BaPeq were calculated according to Equation (3) [52]:

(3) $${\mathrm{BaP}}_{\mathrm{eq}}={\Sigma C}_{s}\times {\;\mathrm{TEF}}_{i}$$

where C_s_ represents the average concentration of an individual PAH.

Humans can encounter the PAHs present in estuarine sediments by oral ingestion and dermal contact. As a result, in addition to calculating the doses of contaminants taken up by human receptors, the incremental lifetime cancer risk by oral ingestion (ILCR_ingestion_) and dermal contact (ILCR_dermal_) was evaluated according to Equations (4) and (5) [49]:

(4) $${\mathrm{ILCR}}_{\mathrm{ingestion}}=\frac{C_{s}\times {\;\mathrm{SF}}_{\mathrm{ingestion}}\times \sqrt[{3}]{\frac{\mathrm{BW}}{70}\;}\times {\;\mathrm{IR}}_{\mathrm{ingestion}}\times \;\mathrm{EF}\;\times \;\mathrm{Dyears}}{\mathrm{BW}\;\times \mathrm{AT}\;\times 10^{6}}{\;}^{\;}$$

(5) $${\mathrm{ILCR}}_{\mathrm{dermal}}=\frac{C_{s}\times {\;\mathrm{SF}}_{\mathrm{dermal}}\times \sqrt[{3}]{\frac{\mathrm{BW}}{70}\;}\times \;\mathrm{AS}\;\times \;\mathrm{AF}\;\times \;\mathrm{ABS}\;\times \;\mathrm{EF}\;\times \;\mathrm{Dyears}}{\mathrm{BW}\;\times \;\mathrm{AT}\;\times 10^{6}}{\;}^{\;}$$

where:

SF_ingestion_ (Kg-day/mg) indicates the oral slope factor.
SF_dermal_ (Kg-day/mg) is the dermal slope factor.
SA (cm^2^/kg) represents the area of dermal contact with the sediment.
AF (mg/cm^2^) is the skin absorption coefficient for the sediment.
ABS is the skin absorption coefficient for contaminants.
AT (years) is the average lifespan.

The parameters used for the calculation of the carcinogenic risk are shown in Table 6.

## 3. Results

### 3.1. PAH Concentrations in Sediment from the Sele River

**Figure 5:**
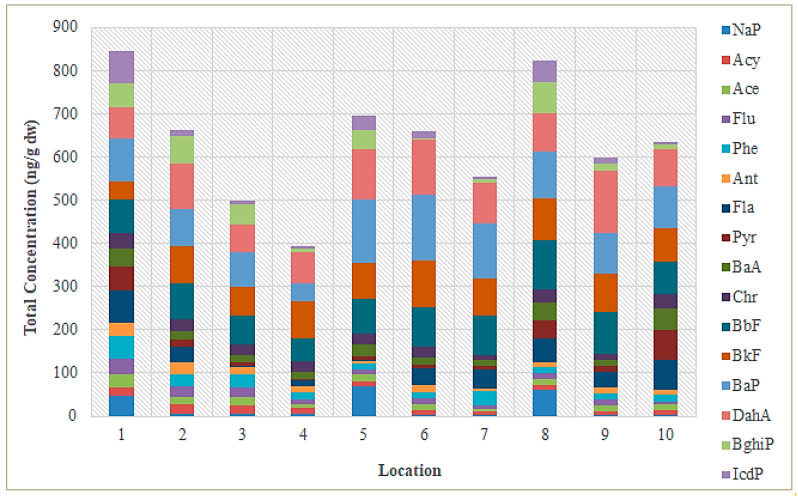
Concentrations of the 16 priority PAHs (ng/g dw) found in sediment samples from the Sele River at 10 sampling locations (April 2021).

**Figure 6:**
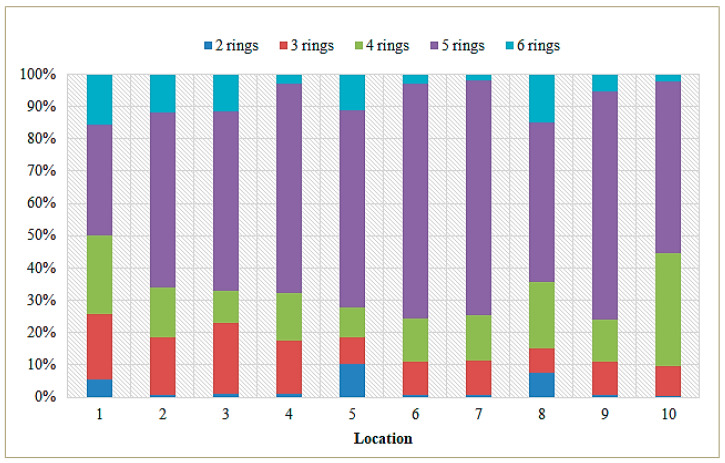
Composition profile of total PAHs in sediment samples from the Sele River.

The concentrations of the 16 USEPA priority PAHs obtained from instrumental analyses of sediment samples taken near the mouth of the Sele River are given in Table S1. In particular, the total concentration of the PAHs ranged from 632.42 ng g^−1^ dw (site 10) to 844.93 ng g^−1^ dw (at site 1), with an average value of 738.68 ng g^−1^ dw. Specifically, the concentrations ranged from 2.23 to 70.64 ng g^−1^ dw with an average value of 36.43 ng g^−1^ dw for PAHs with 2 rings (NaP), from 5.45 to 51.03 ng g^−1^ dw for 3-ring PAHs (Acy, Ace, Flu, Phe, Ant), from 0.70 to 74.6 ng g^−1^ dw for 4-ring PAHs (Flu, Pyr, BaA, Chr), from 39.12 to 154.99 ng g^−1^ dw for 5-ring PAHs (BbF, BkF, BaP, DahA), and from 3.01 to 75.13 ng g^−1^ dw for 6-ring PAHs (BghiP, IcdP). Figure 5 shows the individual concentrations of PAHs detected in sediment samples from different sampling sites. The figure reveals that the highest concentrations of PAHs were found at the mouth of the Sele River (site 1) and 500 m from the mouth in the southerly direction (site 8). The composition profile of PAHs in the sediment is shown in Figure 6. PAHs with 5 rings were found in most test sites at 57.4% of the total PAHs in the sediment.

### 3.2. PAH Concentrations in Sediment from the Sarno River

**Figure 7:**
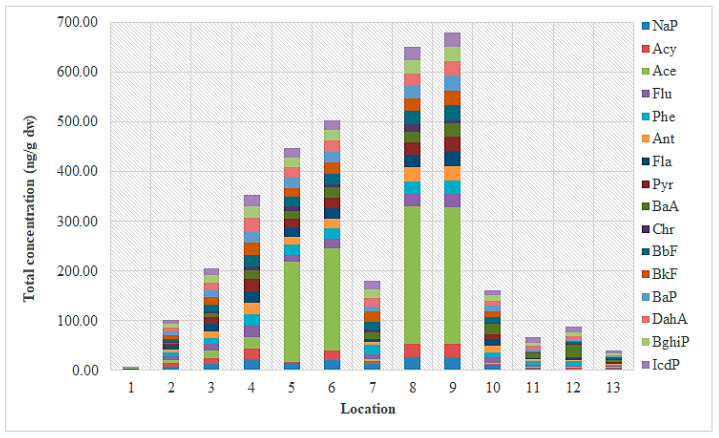
Concentrations of the 16 priority PAHs (ng/g dw) found in sediment samples from the Sarno River at the 13 sampling sites (April 2008).

**Figure 8:**
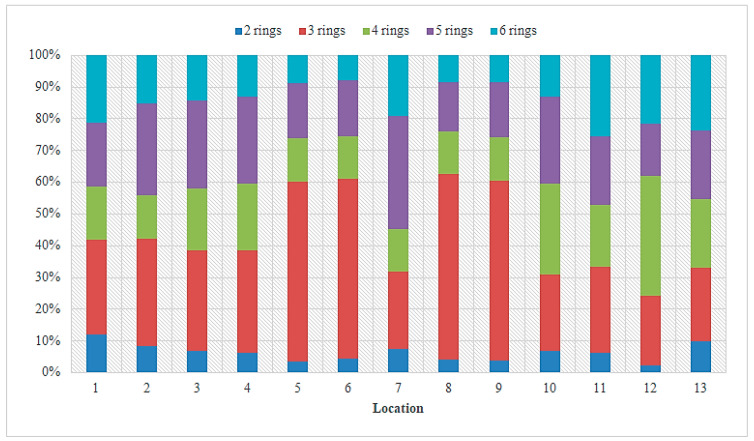
Composition profile of total PAHs in sediment samples from the Sarno River.

Data on PAH concentrations found in the Sarno River are indicated in Table S2 [39]. The total concentration of PAHs in the sediment ranged from 5.2 ng g^−1^ dw at the source of the river (site 1) to 678.6 ng g^−1^ dw at the point 150 m to the west of the mouth (site 9), with an average value of 266.9 ng g^−1^ dw. The measured PAH concentrations ranged from 0.2 to 31.6 ng g^−1^ dw with an average of 9.7 ng g^−1^ dw for 2-ring PAHs (Nap), from 0.2 to 46.3 ng g^−1^ dw for 3-ring PAHs (Acy, Ace, Flu, Phe, Ant), from 0.3 to 47.2 ng g^−1^ dw for 4-ring PAHs (Fla, Pyr, BaA, Chr), from 0.2 to 46.6 ng g^−1^ dw for 5-ring PAHs (BbF, BkF, BaP, DahA), and from 0.5 to 46.7 ng g^−1^ dw for 6-ring PAHs (BghiP, IcdP). Figure 7 shows the individual and total concentrations of PAHs found in the sediment samples taken from the different sampling sites located near the mouth of the Sarno River. The figure indicates an increase in total PAH levels at the sampling point 150 m west of the river mouth (site 8). The composition profile of PAHs in the sediment is shown in Figure 8. Three-ring PAHs were found in most test sites, at a percentage of 47.3% of the total PAH amount in the sediment, followed by 5-ring PAHs at a percentage of 20.6%.

### 3.3. PAH Concentrations in Sediment from the Volturno River

**Figure 9:**
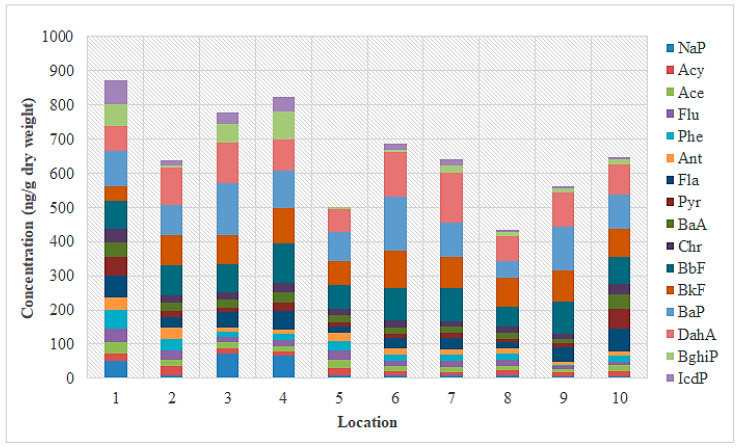
Concentrations of the 16 priority PAHs (ng/g dry weight) found in sediment samples from the Volturno River at 10 sampling locations (April 2018).

**Figure 10:**
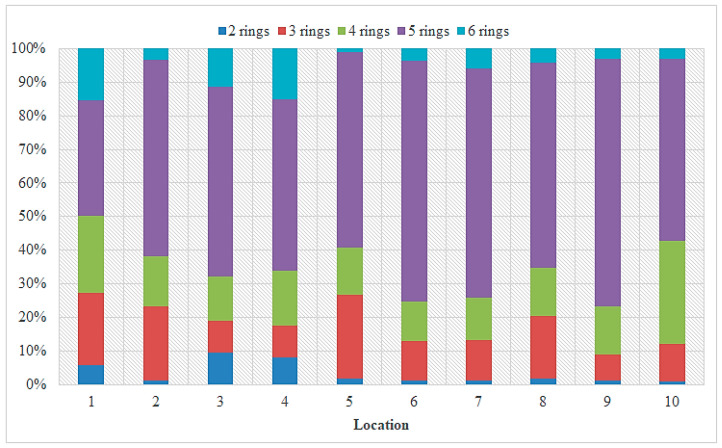
Composition profile of total PAHs in sediment samples from the Volturno River.

Data on individual PAH concentrations found in the Volturno River are given in Table S3, while total concentrations were previously reported [40]. In detail, total concentrations were between 434.8 ng g^−1^ dw (site 8) and 872.1 ng g^−1^ dw (site 1), with an average value of 659.1 ng g^−1^ dw. For 2-ring PAHs (NaP), the levels ranged from 5.3 to 73.8 ng g^−1^ dw with an average value of 24.1 ng g^−1^ dw; for 3-ring PAHs (Acy, Ace, Flu, Phe, Ant), from 42.9 to 186.3 ng g^−1^ dw; for 4-ring PAHs (Fla, Pyr, BaA, Chr), from 61.7 ng g^−1^ dw to 199.7 ng g^−1^ dw; for 5-ring PAHs (BbF, BkF, BaP, DahA), from 262.7 to 507.1 ng g^−1^ dw; and for 6-ring PAHs (BghiP, IcdP), from 17.5 to 133.2 ng g^−1^ dw. Figure 9 shows the individual and total concentrations of PAHs found in the sediment samples taken at the different sampling sites near the mouth of the Volturno River. The figure shows that the highest concentrations of PAHs were found at the mouth of the river (site 1) and 500 m from the mouth in the southerly direction (site 4). The composition profile of PAHs in sediment is shown in Figure 10. Five-ring PAHs were found in most of the test sites at 57,4% of the total PAHs in the sediment.

### 3.4. Evaluation of the Carcinogenic Risk for Human Health from Dermal and Accidental Ingestion Exposure to the PAHs Present in the Sediments of the Surface Waters

To assess the Carcinogenic Risk from exposure to PAHs present in estuarine sediments of the Sele, Volturno, and Sarno Rivers, doses of contaminants taken up by human receptors through the different routes of exposure were evaluated, and the results obtained are reported in Table 7. Particularly, for all three rivers, the doses of each individual PAH and the total doses relating to the entire class of compounds taken up by human receptors through dermal and oral exposure were calculated. The doses from accidental ingestion of PAHs from sediments (Dose_ingestion_) were 7.86 × 10^−4^ mg Kg^−1^/day for the Sele River, 8.13 × 10^−4^ mg Kg^−1^/day for the Volturno River, and 3.31 × 10^−4^ mg Kg^−1^/day for the Sarno River. On the other hand, in relation to the doses of PAHs taken up by skin contact with sediment (Dose_dermal_), the results were 3.23 × 10^−5^, 1.36 × 10^−5^, and 3.35 × 10^−5^ mg Kg^−1^/day for the Sele, Sarno, and Volturno Rivers, respectively.

Moreover, to assess the carcinogenic and mutagenic potencies of PAHs in relation to BaP, the most carcinogenic compound among the PAH considered [10], the average concentrations of individual analytes were converted to the corresponding toxic equivalent concentrations (BaP_eq_). The equivalent toxic concentrations (BaP_eq_) obtained for the Sele, Volturno, and Sarno Rivers are given in Table 7.

Furthermore, the incremental risk of developing lifelong cancer expressed as ILCR was assessed for the exposure to PAHs by ingestion (ILCR_ingestion_) and dermal contact (ILCR_dermal_) [65]. The ILCR_ingestion_ and ILCR_dermal_ values obtained for Sele, Volturno, and Sarno Rivers are shown in Table 8.

The values obtained for carcinogenic risk due to exposure to PAHs by ingestion (ILCR_ingestion_) and dermal contact (ILCR_dermal_) were found to be comparable for the three rivers. According to the USEPA, the ILCR values were interpreted by reference to three ranges, each of which is associated with a risk of carcinogenicity: an ILCR value < 1 × 10^−6^ is associated with a low or zero carcinogenic risk; ILCR values between 1 × 10^−4^ and 1 × 10^−6^ are indicators of a moderate carcinogenic risk; and an ILCR value higher than 1 × 10^−4^ corresponds to a high carcinogenic risk associated with exposure to PAHs in sediment [66,67]. The ILCR_ingestion_ values obtained for the three rivers were found to be much lower than the ILCR_dermal_, indicating that the risk of cancer associated with dermal contact exposure to PAHs present in estuarine sediments may be higher than that associated with accidental ingestion exposure. Specifically, the ILCR_ingestion_ and ILCR_dermal_ values obtained for all sediment samples taken near the mouth of the Sele River ranged between 6.69 × 10^−15^ and 3.11 × 10^−13^ and 7.27 × 10^−8^ and 3.38 × 10^−6^, respectively. The ILCR_ingestion_ and ILCR_dermal_ values obtained for the Sarno River ranged between 2.45 × 10^−15^ and 1.31 × 10^−13^ and 2.66 × 10^−8^ and 1.42 × 10^−6^, respectively. For the Volturno River, the ILCR_ingestion_ values were between 8.23 × 10^−15^ and 3.22 × 10^−13^, while those of ILCR_dermal_ were between 8.95 × 10^−8^ and 3.50 × 10^−6^. Thus, the risk of cancer associated with exposure to PAHs by ingestion of estuarine sediments of the Sele, Sarno, and Volturno Rivers was found to be low at all sampling sites. However, based on the ILCR_dermal_ values obtained, the risk of cancer associated with exposure by dermal contact with the PAHs present in the sediments was found to be moderate (average ILCR_dermal_ for the three rivers of 2.77 × 10^−6^). In addition, the values of the ILCR_ingestion_ and ILCR_dermal_ indices for sediment samples taken at sites with the highest concentrations of PAHs were evaluated. For the Sele and Volturno Rivers, the assessment was carried out at the mouth (site 1), for which total PAH concentrations of 0.8449 and 0.8721 mg Kg^−1^ dw were found, respectively. For the Sarno River, the assessment was carried out at the sampling site 150 m to the west of the estuary (site 9), where a total concentration of PAHs of 0.6792 mg Kg^−1^ dw was found. The results obtained for the three rivers are shown in Table 9.

The ILCR_ingestion_ values obtained for the three rivers at the sampling sites with the highest PAH concentrations were <1 × 10^−6^ (order of 10^−13^), suggesting that the risk of cancer associated with exposure by ingestion of PAHs present in estuarine sediments was low or zero. On the other hand, the ILCR_dermal_ values obtained for the three rivers at the sampling sites with the highest PAH concentrations were in the order of 10^−6^, suggesting that the risk of cancer associated with dermal contact exposure to PAHs present in estuarine sediments was moderate. In fact, as stated also by Cheng et al., ILCR values between 1 × 10^−4^ and 1 × 10^−6^ are associated with a moderate carcinogenic risk [66,67]. Thus, on the basis of the ILCR values obtained by taking into account total PAH concentrations at all sites or considering only the total concentrations recorded at the most polluted sites, the risk of cancer associated with exposure by ingestion was found to be low or zero, but the risk associated with dermal contact exposure of PAHs present in estuarine sediments of the Sele, Volturno, and Sarno Rivers is moderate. Thus, since these areas were previously considered potentially contaminated according to Italian environmental law (D. Lgs. 152/2006), and as stated by Albanese et al., who assessed an incremental lifetime cancer risk higher than 1 × 10^−5^ for the city of Naples [9], a continuous monitoring of potentially hazardous substances is necessary to ensure the protection of public health.

## 4. Conclusions

This paper presents for the first time an assessment of the carcinogenic risk to human health from dermal and ingestion exposure to PAHs present in sediments of the main surface water streams of the Campania Region, southern Italy. The paper also provides information on the concentrations, spatial distribution, and composition profiles of the PAHs detected in sediments collected near the Sele, Sarno, and Volturno River estuaries. The results obtained indicate that the risk of cancer following oral exposure to PAHs in estuarine sediments, expressed as incremental lifetime cancer risk (ILCR_ingestion_), is low, unlike the risk from accidental skin exposure, which was moderate with ILCR_dermal_ values between 1 × 10^−4^ and 1 × 10^−6^. This calls for ongoing assessment of the carcinogenic risk to human health posed by cutaneous and oral exposure to PAHs, as well as constant monitoring of PAH concentrations in surface water sediments in the Campania Region. In conclusion, this study represents a starting point for future studies aimed at assessing the risk of carcinogenicity to human health due to exposure to the PAHs in order to provide support for pollution prevention measures and ecological restoration strategies for rivers, as well as for the preservation of the general well-being.

## Figures and Tables

**Table 1:** Sampling sites with their identification number (ID), location name, and coordinates from the Sele River.

ID	Location	Coordinates	ID	Location	Coordinates
1	Sele River mouth	40°28′55″ N 14°56′33″ E	6	1000 m west	40°28′55″ N 14°55′50″ E
2	500 m north	40°29′04″ N 14°56′14″ E	7	1000 m south	40°28′39″ N 14°55′56″ E
3	500 m west	40°28′55″ N 14°56′12″ E	8	1500 m north	40°29′20″ N 14°55′38″ E
4	500 m south	40°28′47″ N 14°56′16″ E	9	1500 m west	40°28′55″ N 14°55′28″ E
5	1000 m north	40°29′12″ N 14°55′56″ E	10	1500 m south	40°28′30″ N 14°55′38″ E

**Table 2:** Sampling sites with their identification number (ID), location name, and coordinates from the Sarno River.

ID	Location	Coordinates	ID	Location	Coordinates
1	Source of Sarno River	40°48′54.03″ N 14°36′45.36″ E	8	150 m south	40°43′35.68″ N 14°28′02.94″ E
2	Before junction with Alveo Comune	40°46′42.73″ N 14°34′00.48″ E	9	150 m west	40°43′42.25″ N 14°27′59.97″ E
3	After junction with Alveo Comune	40°46′00.34″ N 14°33′10.68″ E	10	150 m north	40°43′49.26″ N 14°27′30.31″ E
4	Sarno River mouth	40°46′10.68″ N 14°28′07.89″ E	11	500 m south	40°43′30.31″ N 14°27′58.94″ E
5	50 m south	40°43′40.11″ N 14°28′06.45″ E	12	500 m west	40°43′42.29″ N 14°27′46.41″ E
6	50 m west	40°43′42.46″ N 14°28′05.03″ E	13	500 m north	40°43′57.85″ N 14°27′48.68″ E
7	50 m north	40°43′45.09″ N 14°28′05.17″ E			

**Table 3:** Sampling sites with their identification number (ID), location name, and coordinates from the Volturno River.

ID	Location	Coordinates	ID	Location	Coordinates
1	Volturno River mouth	40°48′54.03″ N 14°36′45.36″ E	6	1000 m west	40°43′42.46″ N 14°28′05.03″ E
2	500 m north	40°46′42.73″ N 14°34′00.48″ E	7	1000 m south	40°43′45.09″ N 14°28′05.17″ E
3	500 m west	40°46′00.34″ N 14°33′10.68″ E	8	1500 m north	40°43′35.68″ N 14°28′02.94″ E
4	500 m south	40°43′42.62″ N 14°28′07.89″ E	9	1500 m west	40°43′42.25″ N 14°27′59.97″ E
5	1000 m north	40°43′40.11″ N 14°28′06.45″ E	10	1500 m south	40°43′49.26″ N 14°27′59.82″ E

**Table 4:** The 16 Priority PAHs according to the United States Environmental Protection Agency (US EPA) and the World Health Organization (WHO) [20,44].

Name	Abbreviation	Molecular Formula	Number of Rings	Chemical Structure	Molecular Weight
Naphthalene	Nap	C_10_H_8_	2	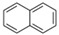	128.2 g mol^−1^
Acenaphthylene	Acy	C_12_H_8_	3	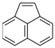	152.2 g mol^−1^
Acenaphthalene	Ace	C_12_H_10_	3	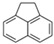	154.2 g mol^−1^
Fluorene	Flu	C_13_H_10_	3	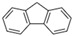	166.2 g mol^−1^
Phenanthrene	Phe	C_14_H_10_	3	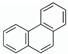	178.2 g mol^−1^
Anthracene	Ant	C_14_H_10_	3	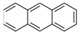	178.2 g mol^−1^
Fluoranthene	Fla	C_16_H_10_	4	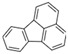	202.3 g mol^−1^
Pyrene	Pyr	C_16_H_10_	4	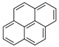	202.3 g mol^−1^
Benzo[a]anthracene	BaA	C_18_H_12_	4	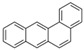	228.3 g mol^−1^
Crysene	Chr	C_18_H_12_	4	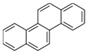	228.3 g mol^−1^
Benzo[b]fluoranthene	BbF	C_20_H_12_	5	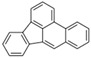	252.3 g mol^−1^
Benzo[k]fluoranthene	BkF	C_20_H_12_	5	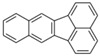	252.3 g mol^−1^
Benzo[a]pyrene	BaP	C_20_H_12_	5	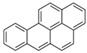	252.3 g mol^−1^
Indeno [123-cd]pyrene	IcdP	C_22_H_12_	6	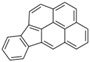	276.3 g mol^−1^
Benzo[ghi]perylene	BghiP	C_22_H_12_	6	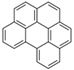	276.3 g mol^−1^
Dibenzo[a,h]anthracene	DahA	C_22_H_14_	5	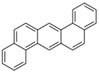	278.3 g mol^−1^

**Table 5:** Equivalent toxicity factors (TEF) of the 16 PAHs [51].

Compound	TEF
Acenaphthalene (Ace)	0.001
Acenaphthylene (Acy)	0.001
Anthracene (Ant)	0.01
Benzo[a]anthracene (BaA)	0.1
Benzo[a]pyrene (BaP)	1
Benzo[b]fluoranthene (BbF)	0.1
Benzo[g,h,i]perylene (BghiP)	0.01
Benzo[k]fluoranthene (BkF)	0.1
Crysene (Chr)	0.01
Dibenzo[a,h]anthracene (DahA)	1
Fluoranthene (Fla)	0.001
Fluorene (Flu)	0.001
Indeno [1,2,3-cd] pyrene (IcdP)	0.1
Naphtalene (Nap)	0.001
Fenanthrene (Phe)	0.001
Pyrene (Pyr)	0.001

**Table 6:** Parameters used for the calculation of doses taken up by human receptors through different routes of exposure.

Parameter	Unit of Measure	Value	References
BW	Kg	70.7	[53]
IR_ingestion_	Kg/days	2.00 × 10^−5^	[54,55]
AT	Years	80	[56,57]
SA_h_	cm^2^	890	[53]
SL_h_	Kg/cm^2^-event	1.00 × 10^−7^	[58]
D_hours_	Hours	0–16/16 h	[59]
D_days_	Days	0–7/7 days	[59]
D_weeks_	Weeks	0–52/52 weeks	[59]
RAF_oral_	-	1	[60]
ED_years_	Years	60	[60]
RAF_derm_^a^	-	0.148	[61]
SF_ingestion_^a^	Kg-day/mg	2.3	[60]
SF_dermal_^a^	Kg-day/mg	25	[62]
EF	(event/day)	1	[63]
SA	cm^2^/kg	5000	[64]
AF	mg/cm^2^	0.04	[64]
ABS	/	0.1	[64]

^a^ expressed in relation to BaP.

**Table 7:** Average total PAH concentrations (C_s_), intakes by accidental ingestion (Dose_ingestion_), and dermal contact (Dose_dermal_) of PAH present in estuarine sediments of the Sele, Volturno, and Sarno Rivers and equivalent toxic concentrations (BaP_eq_).

River	C_s_ (mg Kg^−1^ dw)	Dose_ingestion_ (mg Kg^−1^/day)	Dose_dermal_ (mg Kg^−1^/day)	BaP_eq_ (mg Kg^−1^ dw)
Sele	0.6360	7.86 × 10^−4^	3.23 × 10^−5^	2.23 × 10^−1^
Sarno	0.2677	3.31 × 10^−4^	1.36 × 10^−5^	3.38 × 10^−2^
Volturno	0.6577	8.13 × 10^−4^	3.35 × 10^−5^	2.30 × 10^−1^

**Table 8:** Incremental lifetime cancer risk values (ILCR_ingestion_ and ILCR_dermal_) due to exposure by ingestion and dermal contact from PAHs present in the estuarine sediments of the Sele, Volturno, and Sarno Rivers.

River	ILCR_ingestion_ (mg Kg^−1^ dw)	ILCR_dermal_ (mg Kg^−1^ dw)	ILCR_ingestion/dermal_ [66] Cancerogenic Risk
Sele	3.11 × 10^−13^	3.38 × 10^−6^	If ILCR < 1 × 10^−6^ Low or Zero Risk
Sarno	1.31 × 10^−13^	1.42 × 10^−6^	If 1 × 10^−6^ < ILCR < 1 × 10^−4^ Medium Risk
Volturno	3.22 × 10^−13^	3.50 × 10^−6^	If ILCR >1 × 10^−4^ High Risk

**Table 9:** Incremental lifetime cancer risk values (ILCR_ingestion_ and ILCR_dermal_) due to exposure by ingestion and dermal contact to the highest PAHs levels found in the sediment samples of the Sele, Volturno, and Sarno Rivers.

River	ILCR_ingestion_ (mg Kg^−1^ dw)	ILCR_dermal_ (mg Kg^−1^ dw)	ILCR_ingestion/dermal_ [66] Cancerogenic Risk
Sele	4.14 × 10^−13^	4.50 × 10^−6^	If ILCR < 1 × 10^−6^ Low or Zero Risk
Sarno	3.33 × 10^−13^	3.61 × 10^−6^	If 1 × 10^−6^ < ILCR < 1 × 10^−4^ Medium Risk
Volturno	4.27 × 10^−13^	4.64 × 10^−6^	If ILCR >1 × 10^−4^ High Risk

## Data Availability

Not applicable.

## Supplementary Materials

The following supporting information can be downloaded at: https://www.mdpi.com/article/10.3390/toxics11020172/s1, Table S1: PAH levels (ng g-1 dw) with SD (Standard Deviation) detected in sediment samples from Sele River; Table S2. PAH concentrations (ng g-1 dw) with SD (Standard Deviation) found in sediment samples from the Sarno River (April 2008). Table S3. PAH levels (ng g-1 dry weight) with SD (Standard Deviation) detected in sediment samples from Volturno River.
