# Supplementary material for: Health Risk Assessment of PAHs from Estuarine Sediments in the South of Italy
Source: Toxics. 2023 Feb 13;11(2):172. doi: 10.3390/toxics11020172 (PMC9964163; doi:10.3390/toxics11020172)
Supplement: Supplementary file 1 [file toxics-11-00172-s001.zip › toxics-2175780-supplementary.pdf]

Supplementary Materials

Health Risk Assessment of PAHs from Estuarine Sediments in the South of Italy

Fabiana Di Duca [1], Paolo Montuori [1,*], Ugo Trama [2], Armando Masucci [1], Gennaro Maria Borrelli [1] and Maria Triassi [1]

[1] Department of Public Health, University "Federico II", Via Sergio Pansini n° 5, 80131 Naples, Italy
[2] General Directorate of Health, Campania Region, Centro Direzionale Is. C3, 80143 Naples, Italy
* Correspondence: pmontuor@unina.it

Table S1. PAH levels (ng g^{-1} dw) with SD (Standard Deviation) detected in sediment samples from Sele River

Sampling Location	Concentration (ng g^{-1} dry weight)															
**ID**	**NaP**	**Acy**	**Ace**	**Flu**	**Phe**	**Ant**	**Fla**	**Pyr**	**BaA**	**BbF**	**Chr**	**BkF**	**BaP**	**DahA**	**BghiP**	**IcdP**
1	47.65 ± 3.34	19.01 ± 3.17	30.38 ± 4.52	37.23 ± 3.15	51.03 ± 4.71	31.08 ± 4.07	74.63 ± 7.24	55.25 ± 5.58	42.12 ± 5.89	78.34 ± 8.04	36.26 ± 3.38	39.12 ± 5.61	101.67 ± 6.87	70.65 ± 6.95	55.35 ± 4.51	75.13 ± 6.38
2	5.08 ± 1.28	23.25 ± 4.05	16.27 ± 2.10	24.31 ± 2.63	27.14 ± 2.36	27.55 ± 3.56	36.02 ± 6.39	17.32 ± 3.27	21.42 ± 4.39	84.28 ± 7.43	26.43 ± 2.91	84.23 ± 5.32	86.56 ± 5.19	104.23 ± 8.87	65.56 ± 5.45	12.45 ± 1.98
3	70.64 ± 5.07	11.26 ± 2.88	14.16 ± 1.85	13.17 ± 1.82	12.11 ± 2.25	6.58 ± 1.78	2.43 ± 0.78	9.23 ± 2.15	27.68 ± 3.69	78.64 ± 5.27	24.79 ± 3.47	84.77 ± 5.04	146.46 ± 9.34	115.68 ± 9.10	46.12 ± 3.28	30.89 ± 4.34
4	62.10 ± 4.16	10.25 ± 2.16	13.21 ± 1.57	13.88 ± 1.74	13.73 ± 3.19	12.12 ± 2.50	55.24 ± 6.11	40.20 ± 3.98	43.12 ± 5.12	112.21 ± 9.56	31.11 ± 4.60	98.68 ± 6.27	106.62 ± 8.06	87.69 ± 6.24	72.46 ± 6.80	49.75 ± 6.28
5	5.25 ± 0.95	19.48 ± 3.07	19.45 ± 2.96	23.79 ± 3.41	28.07 ± 3.56	19.09 ± 3.22	0.70 ± 0.36	9.44 ± 1.66	17.33 ± 2.28	66.63 ± 4.10	23.12 ± 3.45	67.10 ± 3.84	81.00 ± 7.36	63.12 ± 6.32	46.23 ± 3.57	10.63 ± 2.25
6	4.32 ± 1.97	10.64 ± 2.04	12.07 ± 1.83	13.35 ± 2.47	14.56 ± 2.05	17.25 ± 2.16	38.21 ± 4.59	8.47 ± 2.14	18.26 ± 3.84	92.47 ± 5.63	23.10 ± 2.71	106.48 ± 7.80	154.99 ± 8.75	126.58 ± 7.82	3.69 ± 1.03	14.35 ± 3.69
7	4.21 ± 1.18	7.31 ± 1.85	13.02 ± 2.24	14.04 ± 3.06	14.20 ± 1.97	12.75 ± 1.90	38.21 ± 4.25	12.11 ± 3.18	14.95 ± 3.67	95.85 ± 7.14	14.48 ± 2.96	87.81 ± 6.65	96.45 ± 6.30	143.36 ± 6.41	17.12 ± 3.22	14.33 ± 2.47
8	4.50 ± 1.75	14.34 ± 2.79	9.07 ± 1.40	12.26 ± 2.45	15.24 ± 3.12	13.69 ± 2.39	13.12 ± 3.12	5.05 ± 1.64	16.25 ± 4.17	53.86 ± 6.80	23.35 ± 3.64	84.37 ± 5.47	44.04 ± 3.27	71.20 ± 7.80	8.13 ± 2.56	3.79 ± 1.10
9	3.53 ± 0.84	8.46 ± 1.73	5.19 ± 1.17	6.96 ± 1.83	33.09 ± 4.18	6.09 ± 2.47	45.85 ± 5.61	6.64 ± 2.27	13.82 ± 2.52	91.02 ± 7.93	11.43 ± 2.88	87.76 ± 7.05	126.46 ± 8.18	95.34 ± 8.63	7.79 ± 1.36	2.65 ± 0.97
10	2.23 ± 0.96	12.60 ± 3.14	14.14 ± 2.89	5.45 ± 1.79	16.08 ± 1.75	10.54 ± 3.04	70.07 ± 6.50	69.19 ± 7.22	48.87 ± 6.81	76.00 ± 4.55	33.23 ± 4.27	76.20 ± 5.10	98.34 ± 7.44	85.90 ± 8.46	10.56 ± 2.20	3.01 ± 1.34

Table S2. PAH concentrations (ng g^{-1} dw) with SD (Standard Deviation) found in sediment samples from the Sarno River (April 2008) [39].

Sampling Location	Concentration (ng g^{-1} dry weight)															
ID	NaP	Acy	Ace	Flu	Phe	Ant	Fla	Pyr	BaA	BbF	Chr	BkF	BaP	DahA	BghiP	IcdP
1	0.68 ± 0.35	0.28 ± 0.11	0.20 ± 0.12	0.24 ± 0.08	0.59 ± 0.21	0.36 ± 0.15	0.37 ± 0.15	0.18 ± 0.07	0.22 ± 0.10	0.23 ± 0.12	0.18 ± 0.05	0.30 ± 0.14	0.20 ± 0.12	0.39 ± 0.14	0.71 ± 0.39	0.48 ± 0.21
2	8.41 ± 2.36	6.26 ± 1.19	6.19 ± 1.31	7.27 ± 1.48	7.44 ± 1.35	7.16 ± 1.35	7.08 ± 1.63	4.34 ± 1.22	1.39 ± 0.48	7.33 ± 1.17	1.15 ± 0.48	7.26 ± 1.57	7.05 ± 1.87	7.65 ± 1.25	7.59 ± 1.12	7.69 ± 1.45
3	14.02 ± 3.19	11.36 ± 2.30	15.14 ± 2.06	12.14 ± 2.62	12.01 ± 2.25	14.28 ± 2.18	14.38 ± 2.71	14.39 ± 3.41	7.26 ± 1.07	13.04 ± 2.35	4.25 ± 1.25	14.49 ± 2.69	14.26 ± 2.73	14.89 ± 2.48	15.02 ± 2.28	14.22 ± 2.09
4	22.27 ± 4.93	22.10 ± 3.64	23.20 ± 3.48	22.29 ± 3.19	22.71 ± 2.56	23.25 ± 3.38	24.04 ± 3.68	23.31 ± 4.64	17.75 ± 2.30	22.09 ± 3.48	9.02 ± 2.32	25.13 ± 3.87	21.71 ± 3.70	27.85 ± 3.04	22.31 ± 3.45	23.32 ± 2.37
5	15.37 ± 3.25	1.77 ± 0.63	200.68 ± 8.92	14.55 ± 2.37	20.81 ± 3.69	15.90 ± 1.69	17.70 ± 2.06	17.67 ± 2.31	15.78 ± 1.87	20.21 ± 2.56	9.15 ± 1.78	17.44 ± 3.15	20.49 ± 2.97	20.27 ± 3.33	19.44 ± 2.64	19.37 ± 2.18
6	21.67 ± 4.89	18.35 ± 3.88	205.84 ± 9.13	19.18 ± 2.91	20.51 ± 2.86	20.26 ± 3.24	21.17 ± 3.55	21.06 ± 2.68	20.07 ± 2.49	21.14 ± 3.69	5.90 ± 1.36	23.21 ± 2.78	21.31 ± 3.46	22.47 ± 4.16	20.57 ± 3.81	19.02 ± 2.60
7	13.41 ± 2.37	5.44 ± 1.27	3.65 ± 1.02	9.44 ± 1.40	18.86 ± 3.04	6.47 ± 1.15	3.53 ± 1.27	2.75 ± 1.15	12.49 ± 2.05	15.11 ± 1.44	5.82 ± 0.98	22.54 ± 2.39	9.60 ± 2.05	17.09 ± 1.98	17.02 ± 2.66	17.21 ± 1.78
8	26.46 ± 4.65	27.32 ± 4.03	276.58 ± 7.57	24.91 ± 3.85	25.47 ± 3.97	27.55 ± 3.60	25.32 ± 3.58	25.78 ± 3.47	20.98 ± 2.31	27.20 ± 3.61	14.59 ± 1.17	25.39 ± 3.97	25.92 ± 4.23	23.48 ± 3.11	26.17 ± 4.03	28.21 ± 2.27
9	26.83 ± 5.68	26.24 ± 3.85	274.99 ± 9.36	26.54 ± 4.23	27.73 ± 4.32	28.17 ± 3.54	31.02 ± 4.82	28.27 ± 4.56	27.67 ± 3.82	29.02 ± 2.87	6.12 ± 1.78	29.36 ± 4.02	30.52 ± 4.51	29.03 ± 2.18	29.04 ± 3.28	28.61 ± 3.15
10	11.31 ± 2.66	2.33 ± 0.72	1.74 ± 0.50	10.74 ± 1.47	10.47 ± 1.23	13.29 ± 1.02	13.46 ± 2.30	9.11 ± 1.38	21.30 ± 3.02	10.55 ± 1.94	2.88 ± 0.45	11.04 ± 2.32	11.21 ± 2.33	11.16 ± 1.25	11.04 ± 1.59	10.29 ± 1.77
11	4.12 ± 3.07	2.12 ± 0.58	1.45 ± 0.71	3.58 ± 1.09	8.65 ± 1.17	2.25 ± 0.63	2.46 ± 1.01	0.96 ± 0.32	8.04 ± 1.25	1.99 ± 0.58	1.45 ± 0.69	1.73 ± 0.86	3.45 ± 1.31	7.37 ± 1.06	6.07 ± 1.23	10.81 ± 2.05
12	2.08 ± 0.95	2.66 ± 1.01	1.86 ± 0.66	2.59 ± 0.97	9.18 ± 2.03	2.85 ± 0.96	3.32 ± 1.23	2.18 ± 0.59	24.19 ± 4.11	3.25 ± 1.20	3.30 ± 0.75	2.32 ± 1.28	2.25 ± 0.95	6.39 ± 1.25	7.47 ± 1.20	11.38 ± 1.54
13	4.02 ± 1.54	1.35 ± 0.60	1.36 ± 0.47	2.13 ± 0.82	3.29 ± 0.76	1.25 ± 0.49	2.02 ± 0.75	1.18 ± 0.35	4.26 ± 1.63	4.26 ± 1.75	1.24 ± 0.58	1.14 ± 0.43	1.36 ± 0.69	1.97 ± 0.88	4.48 ± 1.15	5.06 ± 0.95

Table S3. PAH levels (ng g-1 dry weight) with SD (Standard Deviation) detected in sediment samples from Volturno River [40].

Sampling Location	Concentration (ng g-1 dry weight)															
**ID**	**NaP**	**Acy**	**Ace**	**Flu**	**Phe**	**Ant**	**Fla**	**Pyr**	**BaA**	**BbF**	**Chr**	**BkF**	**BaP**	**DahA**	**BghiP**	**IcdP**
1	50.77 ± 4.58	22.05 ± 3.75	33.73 ± 3.91	40.17 ± 4.19	54.73 ± 5.27	35.67 ± 3.03	62.74 ± 4.12	56.64 ± 4.06	42.40 ± 3.89	81.43 ± 4.62	38.01 ± 2.43	42.25 ± 3.84	104.74 ± 4.52	73.55 ± 4.20	62.27 ± 4.31	70.96 ± 3.15
2	8.11 ± 2.12	26.62 ± 4.18	19.22 ± 2.39	27.22 ± 2.30	33.43 ± 4.15	32.11 ± 2.99	30.79 ± 2.41	19.10 ± 1.27	23.46 ± 1.97	87.49 ± 3.19	23.04 ± 2.64	87.31 ± 4.10	89.66 ± 3.49	107.35 ± 4.12	6.18 ± 1.05	15.46 ± 2.24
3	73.78 ± 8.96	14.47 ± 2.33	17.67 ± 3.60	16.38 ± 2.54	15.56 ± 2.34	10.06 ± 2.08	46.22 ± 3.89	12.53 ± 2.15	23.31 ± 2.14	81.76 ± 4.36	20.78 ± 3.10	87.68 ± 2.23	149.56 ± 5.69	118.78 ± 4.76	56.34 ± 3.51	32.64 ± 3.21
4	65.27 ± 5.39	13.63 ± 1.89	16.17 ± 2.88	16.81 ± 3.18	17.25 ± 3.10	14.51 ± 1.45	54.30 ± 2.58	23.58 ± 2.69	29.70 ± 2.38	115.15 ± 5.48	28.33 ± 3.95	101.98 ± 3.75	109.66 ± 4.50	90.96 ± 3.41	81.92 ± 4.03	43.05 ± 2.26
5	8.72 ± 1.67	22.59 ± 3.96	22.72 ± 3.99	26.90 ± 2.49	28.95 ± 3.62	24.09 ± 2.16	18.94 ± 1.78	12.27 ± 1.44	20.23 ± 2.06	69.73 ± 3.47	18.65 ± 2.50	70.32 ± 4.69	84.00 ± 3.19	66.22 ± 3.17	5.39 ± 1.10	<LOQ
6	7.49 ± 1.34	13.52 ± 2.55	15.11 ± 2.48	16.35 ± 1.93	17.41 ± 2.13	18.55 ± 2.94	29.38 ± 3.12	11.69 ± 1.07	20.15 ± 2.28	95.85 ± 3.89	19.32 ± 2.32	109.78 ± 3.01	157.86 ± 4.58	129.76 ± 4.23	7.01 ± 1.43	17.33 ± 1.87
7	7.36 ± 2.03	10.26 ± 2.12	16.22 ± 2.92	17.19 ± 2.28	18.49 ± 2.52	15.77 ± 1.60	32.30 ± 3.25	14.93 ± 2.05	17.63 ± 1.49	98.91 ± 4.18	16.03 ± 2.14	90.79 ± 4.46	99.51 ± 3.92	146.57 ± 5.04	20.99 ± 3.21	17.23 ± 2.05
8	7.81 ± 1.23	17.35 ± 3.27	12.18 ± 1.35	15.70 ± 1.44	18.92 ± 1.66	17.32 ± 2.38	16.23 ± 1.30	8.64 ± 1.19	19.00 ± 2.14	56.78 ± 2.49	17.88 ± 3.09	87.53 ± 5.18	47.04 ± 2.15	74.20 ± 4.25	12.15 ± 1.48	6.10 ± 1.02
9	6.79 ± 1.20	11.85 ± 1.19	8.20 ± 1.32	9.90 ± 1.15	3.65 ± 1.45	9.37 ± 1.13	42.55 ± 3.68	9.30 ± 1.77	14.45 ± 1.78	94.20 ± 4.70	13.95 ± 1.49	90.63 ± 4.13	129.61 ± 4.26	98.44 ± 4.89	12.18 ± 1.27	5.38 ± 0.95
10	5.30 ± 1.36	15.76 ± 1.87	17.30 ± 2.15	8.65 ± 1.09	18.29 ± 2.27	12.87 ± 1.64	66.43 ± 4.14	59.65 ± 3.09	40.34 ± 3.28	79.04 ± 4.55	32.02 ± 3.05	79.98 ± 4.49	101.65 ± 4.37	88.95 ±3.36	14.14 ± 2.25	5.93 ± 1.41
